# Synthesis of Thermo-Responsive Polymer via Radical (Co)polymerization of *N*,*N*-Dimethyl-α-(hydroxymethyl)acrylamide with *N,N*-Diethylacrylamide

**DOI:** 10.3390/polym8100374

**Published:** 2016-10-20

**Authors:** Yasuhiro Kohsaka, Yoshiaki Tanimoto

**Affiliations:** Faculty of Textile Science and Technology, Shinshu University, Nagano 386-8567, Japan; 16fs515j@shinshu-u.ac.jp

**Keywords:** α-functionalized acrylamide, temperature-responsive polymer, lower critical solution temperature, hydrophilic monomer, radical polymerization

## Abstract

α-Functionalized acrylamides have not been considered as an effective monomer design due to their poor polymerizability, although the analogues, α-functionalized acrylates, are attractive monomers of which polymers exhibit characteristic properties. In this article, we report the first example of radical polymerization of α-functionalized *N*,*N*-disubstituted acrylamide affording thermo-responsive hydrophilic polymers. *N*,*N*-dimethyl-α-(hydroxymethyl)acrylamide (DMαHAA) was (co)polymerized with *N*,*N*-diethylacrylamide (DEAA). Although the homopolymerization did not afford a polymeric product, the copolymerizations with various feed ratios yielded a series of the copolymers containing 0%–65% of DMαHAA units. The obtained copolymers exhibited a lower critical solution temperature (LCST) in water; the cloud points (*T*_c_s) were linearly elevated as the contents of DMαHAA units from 32 to 64 °C, indicating that DMαHAA functioned as a more hydrophilic monomer than DEAA. The linear relationship between *T*_c_ and DMαHAA content suggests that the homopolymer, poly(DMαHAA), should have *T*_c_ at ca. 80 °C, although it is not available by direct radical homopolymerization.

## 1. Introduction

As acrylamides have tunable hydrophilicity/hydrophobicity by one or two *N*-substituents, various thermo-responsive polymers based on acrylamides such as *N*-isopropylacrylamide [1,2,3,4,5] and *N*,*N*-diethylacrylamide (DEAA) [6,7,8] have been developed. On the other hand, *N*,*N*-disubstituted methacrylamides are poorly polymerizable both in radical and anionic polymerization [9,10,11,12,13], although they have acrylamide skeletons, where the vinylidene groups should be activated with the electron-withdrawing carbonyl groups. A difference between their typical monomer, *N*,*N*-dimethylmethacrylamide (DMMA), and the polymerizable analogue, *N*,*N*-dimethylacrylamide (DMAA), is the existence of an α-methyl group. Therefore, the α-methyl group is attributed to the poor polymerizability. In fact, spectral analyses [14,15] and modified neglect of differential overlap (MNDO) calculations [15] of DMMA suggested a twisted conformation between the vinyl and carbonyl groups; if they are in the same plane, the α-methyl group causes the steric repulsion against the *N*-methyl group in the cisoid form, while the *N*-substituent creates a similar repulsion to the vinylic hydrogen atom in the transoid form. Therefore, DMMA and other *N,N*-disubstituted methacrylamides had been categorized as unconjugated vinyl monomers.

Recently, we have been interested in the polymerization chemistry of α-functionalized acrylates, because the α-functionality affects both the polymerization behavior and the properties of the resulting polymer [16,17,18,19,20]. For example, ethyl α-(aminomethyl)acrylate forms an intramolecular hydrogen bond with the carbonyl group, which reduces the nucleophilicity of the amino group and stabilizes the monomer against the ester-amide exchange reaction [19]. The radical polymerization takes place through the unique mechanism accompanying the in situ modification of the pendant amino groups. The resulting polymer exhibits pH/temperature responsivity in acidic aqueous media.

Along the extended line of this concept, we have an interest in the polymerization of α-functionalized acrylamides. Unfortunately, however, the syntheses of α-functionalized *N*-alkylacrylamides are difficult due to the presence of the acidic amide proton. On the other hand, the preparations of α-functionalized *N*,*N*-dialkylacrylamides are relatively facile to prepare [21]. As previously mentioned, *N*,*N*-dialkylmethacrylamides are not polymerizable, and thus there are no reports on the polymerization of α-functionalized *N*,*N*-dialkylacrylamides, although the α functionality may affect the polymerizability. Herein, we report the polymerization of the hydroxy-functionalized monomer *N*,*N*-dimethyl-α-(hydroxymethyl)acrylamide (DMαHAA), and the thermo-responsive properties of the resulting polymer.

## 2. Experimental Section

### 2.1. Materials

DEAA and DMAA were kind gifts from KJ Chemicals Co., Ltd. (Tokyo, Japan). These monomers were used after distillation under reduced pressure. 2,2′-Azobis(isobutyronitrile) (AIBN) was recrystallized in hexane before use. Wakogel C-400HG (Wako Pure Chemical Industries, Osaka, Japan) was used as a silica gel for chromatography. Other solvents and reagents were used as received.

### 2.2. Instruments

^1^H NMR spectra were recorded in CDCl_3_ (Nacalai Tesque, Kyoto, Japan) on an AVANCE 400 (Bruker, Billerica, MA, USA) spectrometer. Chemical shifts in ^1^H NMR spectra were referred to the signal of tetramethylsilane (TMS). Molecular weight and its distributions were determined at 40 °C by size-exclusion chromatography (SEC) on an EXTREMA chromatograph (JASCO Co., Tokyo, Japan) equipped with two SEC columns (PL-gel, Mixed C (300 mm_7.5 mm), Polymer Laboratories), using tetrahydrofuran (THF) as an eluent (flow rate = 0.8 mL·min^−1^), and calibrated against standard polystyrene (PS) samples (TSK-gel oligomer kit, Tosoh, Tokyo, Japan, *M*_n_: 1.03 × 10^6^, 3.89 × 10^5^, 1.82 × 10^5^, 3.68 × 10^4^, 1.36 × 10^4^, 5.32 × 10^3^, 3.03 × 10^3^, 8.73 × 10^2^) and detected with UV (UV-4070, JASCO Co., Tokyo, Japan) and RI (RI-4030, JASCO Co., Tokyo, Japan) detectors. Purities of monomers were determined from the gas chromatogram (GC) recorded on a GC-2014 (Shimadzu, Kyoto, Japan) equipped with an SH-Rtx-5 capillary column (Shimadzu, Kyoto, Japan).

### 2.3. Synthesis of N,N-Dimethyl-α-(hydroxymethyl)acrylamide [21]

To a solution of diazabicyclo[2.2.2]octane (DABCO, Tokyo Chemical Industry Co., Ltd., Tokyo, Japan, 18.2 g, 0.162 mmol) in water (28 mL) was added a solution of phenol (7.67 g, 81.5 mmol) in *tert*-butanol (12 mL). DMAA (67.0 mL, 0.650 mol) was added, and the reaction mixture was heated at 65 °C for five days. After concentration, acetone-hexane (*v*/*v* = 4/1) cosolvent and silica gel (130 g) was added and the reaction mixture was stirred for several hours and then filtered. The filtrate was concentrated, and the residual product was purified by distillation under reduced pressure (132 °C/56.0 Pa–135 °C/61.0 Pa) to afford colorless oil (12.2 g). DMαHAA: yield 14.5%; purity 99.7%, ^1^H NMR spectrum: δ/ppm 5.53–5.52 (*m*, 1H, CH*H*=), 5.23 (*dd*, *J*_1_ = *J*_2_ = 8.8 Hz, 1H, C*H*H=), 4.30 (*s*, 2H, allylic proton), 3.10 (*s*, 3H, *N*–CH_3_), 3.01 (*s*, 3H, *N*–CH_3_), 2.91 (*br*, 1H, OH).

### 2.4. Radical Polymerization

A typical example (Table 1, Run 5): In a glass test tube, a mixture of DMαHAA (210 mg, 1.65 mmol), DEAA (190 mg, 1.53 mmol) and AIBN (5.1 mg, 31 μmol) was dissolved in 1,4–dioxane (3.0 mL). The reaction mixture degassed using a freeze–pump–thaw cycle three times. Nitrogen gas was then introduced into the test tube. The reaction mixture was heated at 65 °C for 12 h. A small portion of the reaction mixture was sampled to estimate the monomer conversion. The reaction mixture was then poured into hexane (200 mL) and the precipitate was collected, washed with hexane, and dried in vacuo to give a copolymer (311 mg), as a white solid.

### 2.5. Observation of Cluod Point

A typical example: The polymer obtained in Run 2 (5 mg) was dissolved in distilled water (1 mL) and the solution was heated on hot-plate with a rate of temperature increase as 1 °C·min^−1^. The cloud point was visually examined as the temperature where solution became cloudy.

### 2.6. Density Functional Theory (DFT) Calculations

All DFT calculations were performed using Spartan’16 (Wavefunction Inc., Irvine, CA, USA) on a laptop PC (Inspiron 15, DELL, Round Rock, TX, USA) equipped with an Intel core i5-5200U processer (2.20 GHz) with an 8.00 GB RAM. The most stable conformation was searched and optimized with molecular mechanics (MM) based on MVFF94 force field before DFT calculation. B3LYP/6-31G* were used for geometry optimization and the subsequent single point calculation on DFT calculation.

## 3. Results and Discussion

### 3.1. Attempted Homopolymerization of DMαHAA

**Scheme 1:**
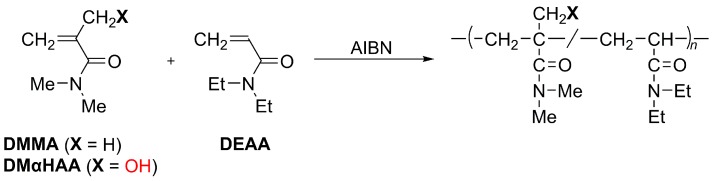
Radical (co)polymerization of DMMA and DMαHAA with DEAA.

DMαHAA was prepared through the Baylis-Hillman reaction between DMAA and formaldehyde according to the literature [21]. The radical polymerization of DMαHAA was conducted in 1,4-dioxane with AIBN at 65 °C (Scheme 1, Table 1, Run 2). For the control, DMMA and DEAA were polymerized in a similar manner (Runs 1 and 3) in order to examine the effects of the hydroxy group and the α-substituent on polymerization. DMαHAA and DMMA did not yield polymeric products, whereas DEAA afforded the respective polymer.

**Figure 1:**
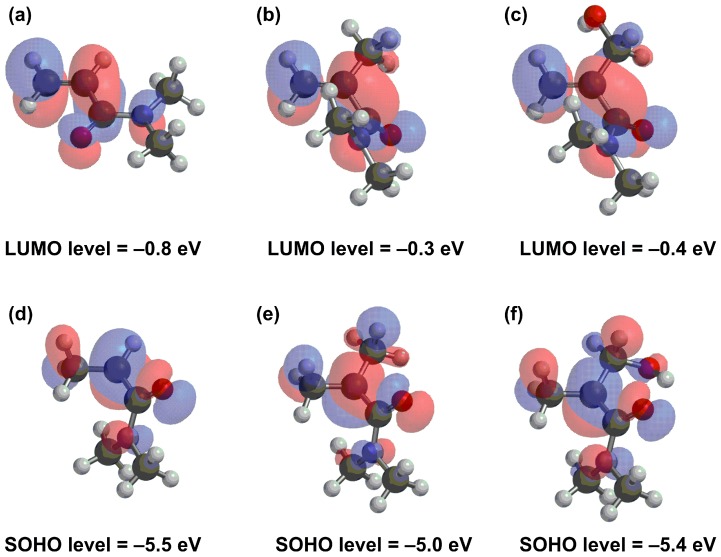
LUMOs of (**a**) DMAA, (**b**) DMMA and (**c**) DMαHAA, and SOHOs of the respective radicals to (**d**) DMAA, (**e**) DMMA and (**f**) DMαHAA, by DFT calculation (B3LYP/6-31G*).

As mentioned in the introduction, *N*,*N*-disubstituted methacrylamides are not polymerizable due to the twisted acrylamide skeletons. Consequently, these results are reasonable. In fact, the optimized structures of both DMαHAA and DMMA by density functional theory (DFT) calculations exhibit twisted, non-planar acrylamide skeletons (Figure 1b,c). Nevertheless, importantly, the lowest unoccupied molecular orbitals (LUMOs) of DMαHAA and DMMA spread over the acrylamide skeleton similarly to that of DMAA, a simplified model monomer of DEAA (Figure 1a). This suggests that these monomers have capacities to accept the radical attack to yield the respective monomer-end radicals, despite the non-planar acrylamide skeletons described in the classical discussion. It should be noted that DMAA has a lower LUMO level than others, and thus it should have higher reactivity than the other two monomers. The singly occupied highest orbitals (SOHOs) of the model molecules of resulting radicals were also calculated (Figure 1d–f). These three monomers have similar orbital shapes. For the respective monomers, the effects of the steric repulsion of substituents on the planarity of acrylamide skeletons had been discussed previously. On the other hand, the radicals have sp^3^ carbons and thus the steric repulsions of substituents are not significant. The presence of the α-substituent, of course, may influence the propagation rate and entropy of polymerization, but these calculation results implied that DMαHAA and DMMA should intrinsically have the potential to polymerize. Presumably, the loss of polymerizability might be caused by the short lifetime of the respective radicals or the large entropy of polymerization. Nevertheless, the results of the DFT calculation suggest that these monomers may undergo copolymerization with appropriate polymerizable monomers.

### 3.2. Copolymerization of DMαHAA with DEAA

**Figure 2:**
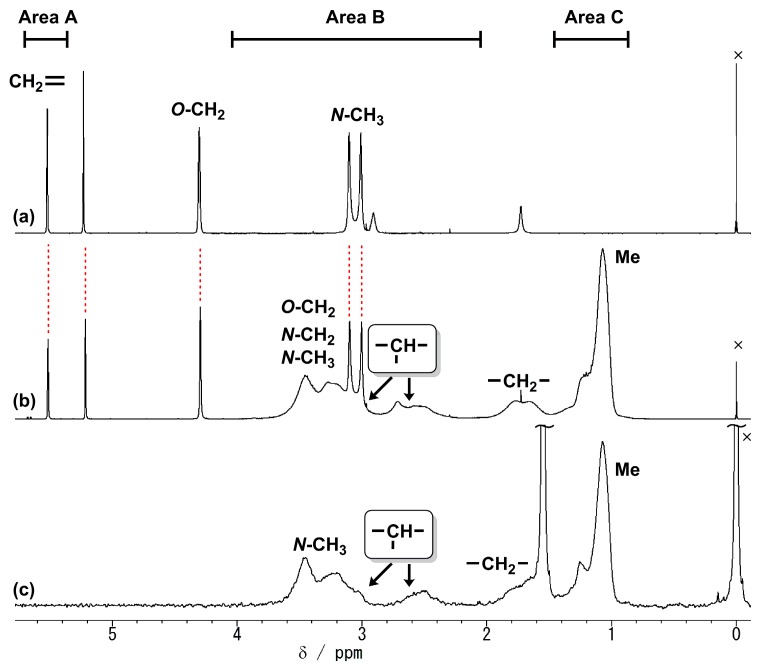
^1^H NMR spectra of (**a**) DMαHAA; (**b**) poly(DMαHAA-*co*-DEAA) obtained from Run 5; and (**c**) poly(DEAA) (400 MHz, CDCl_3_, 25 °C). **×**: Tetramethylsilane.

The copolymerizations of DMMA and DMαHAA with an equimolar amount of DEAA were conducted in a similar way to the homopolymerization. In both cases (Runs 4 and 5), the respective copolymers were obtained as expected. Figure 2b shows the ^1^H NMR spectra of the obtained copolymer of DMαHAA and DEAA, accompanying the monomer (DMαHAA) and poly(DEAA). Since the polymer included the unreacted monomer and the ^1^H NMR signals of each monomer unit were overlapped, the polymer composition was determined as follows:

- Area A (5.75–5.35 ppm) includes the vinylidene signal of the unreacted monomer.
- Area B (4.05–2.05 ppm) includes the *N*–CH_2_ and main-chain CH signals in DEAA units, the *N*–CH_3_ and *O*–CH_2_ signals in DMαHAA units, and the *N*–CH_3_ signals of unreacted DMαHAA.
  If we set the content of DMαHAA units as *x* (%), the following equation can be established:
  [Area B] − 6 × [Area A] _(*N-*CH_3_ of unreacted DMαHAA)_ = 4*x*_(*N-*CH_2_)_ + *x*_(CH)_ + 6 × (100 − *x*) _(*N-*CH_3_)_ + 2 × (100 – *x*) _(*O-*CH_2_)_ (1)
- Area C (1.45–0.85 ppm) includes the *N*–CH_2_C*H*_3_ signal in DEAA units, and thus the following equation can be established:
  [Area C] = 6*x*_(_*_N_*_-CH_2_C*H*_3___)_ (2)

The intensity integrations of each area were inserted in Equations (1) and (2), and the simultaneous equation was solved to give the content of DMαHAA units (*x* = 20%).

The resulting polymers of the copolymerization of DMMA and DEAA had similar compositions, i.e., 23% of DMAA units against 50% of the feed ratio. Therefore, the effect of the hydroxy group on the polymerization behavior was not significant.

The polymerizations were also examined in other solvents such as CHCl_3_ (Run 6), DMF (Run 7), ethanol (Run 8), and water (Run 9). While the polymerizations in aprotic solvents afforded similar results with ca. 20% of DMαHAA unit contents, those in protonic solvents such as water and ethanol resulted in significantly different results. In ethanol, no polymeric product was obtained, whereas the homopolymerization of DEAA almost took place in water. Careful discussions are required to explain these results, but presumably the hydrogen bond formation of the protonic solvent molecules to the hydroxy groups of DMαHAA might increase the steric hindrance around the vinylidene group to prevent the polymerization of DMαHAA.

### 3.3. Syntheses of Thermo-Responsive Polymers with Tunable Cloud Points

**Figure 3:**
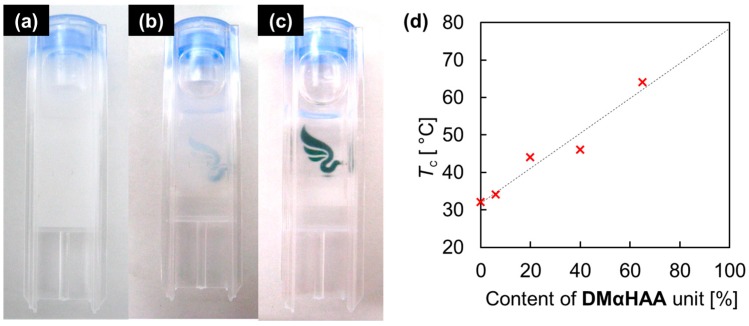
Photographs of poly(DMαHAA-*co*-DEAA), obtained in Run 5, in water (1 wt %): (**a**) at 50 °C; (**b**) at 44 °C; and (**c**) at 25 °C; (**d**) Plots of content of DMαHAA unit versus *T*_c_.

As is well known, poly(DEAA) exhibited a lower critical solution temperature (LCST) around 32 °C. Similarly, the copolymer of DEAA and DMαHAA obtained in Run 4 displayed the cloud point (*T*_c_) at 44 °C, as shown in Figure 3b and Video S1 (available as Supplementary Materials on web), indicating that the incorporation of the hydrophilic hydroxy group increased the *T*_c_. In order to tune the *T*_c_, a series of copolymers with different compositions was prepared (Table 2, Runs 3, 5, 10–12). The molecular weights of the resulting copolymers became lower as the feed ratio of the DMαHAA increased, probably because of the poor homopolymerizablity. It should be noted that the content of the DMαHAA units reached 65% in Run 12, suggesting the existence of the homosequence of DMαHAA-DMαHAA. This means that DMαHAA has slight homopolymerizablity, although the homopolymerization did not yield a polymeric product, probably due to the fast termination.

The *T*_c_s were linearly increased as the contents of the DMαHAA units, as shown Figure 3d. If the linear relationship is maintained in the extended region, the homopolymer poly(DMαHAA) should have a *T*_c_ around 80 °C, although it could not be prepared by the direct polymerization of DMαHAA.

## 4. Conclusions

Although *N*,*N*-disubstituted acrylamides had been categorized as unconjugated monomers due to their twisted conformation and thus as not polymerizable, the DFT calculations show the expectation of the potential polymerizability. In fact, DMαHAA did not yield the homopolymer but allowed radical copolymerization with DEAA. The hydrophilicity of the hydroxy group increased the *T*_c_, and the linear relationship between the content of the DMαHAA unit and *T*_c_ provides the presumption of the *T*_c_ of poly(DMαHAA) around 80 °C. Importantly, the analogue, poly(*N*,*N*-dimethylacrylamide), is a water-soluble polymer and displays no *T*_c_. Although the hydroxy group is hydrophilic, the α-substitution did not simply enhance the hydrophilicity to the polymer but gave the thermo-responsivity. This fact clearly shows the importance of α-functionalization. Now the authors are attempting controlled polymerization of these α-functionalized *N*,*N*-disubstituted acrylamides, as they can accept a radical addition if the radical has a long lifetime. The results will be reported elsewhere soon.

## Figures and Tables

**Table 1:** Radical (co)polymerizations of DMMA (*N*,*N*-dimethylmethacrylamide) and DMαHAA (*N*,*N*-dimethyl-α-(hydroxymethyl)acrylamide) with DEAA (*N*,*N*-diethylacrylamide).

Run ^1^	M_1_ (mmol)	DEAA (mmol)	Solvent	Yield (%)	Composition ^2^		*M*_n_^3^	*M*_w_/*M*_n_^3^
					M_1_	M_2_		
1	DMMA (1.90)	-	- (bulk)	0	No polymeric products			
2	DMαHAA (2.00)	-	- (bulk)	0	No polymeric products			
3	-	(3.12)	1,4-dioxane	78	0	100	25,000	1.71
4	DMMA (1.60)	(1.49)	1,4-dioxane	35	23	77	5,500	1.44
5	DMαHAA (1.65)	(1.53)	1,4-dioxane	57	20	80	1,400	2.90
6	DMαHAA (1.50)	(1.57)	CHCl_3_	48	24	76	3,400	1.57
7	DMαHAA (1.57)	(1.50)	DMF	71	25	75	4,600	1.87
8	DMαHAA (1.51)	(1.49)	Ethanol	0	No polymeric products			
9 ^4^	DMαHAA (1.56)	(1.55)	H_2_O	33.1	4	96	25,000	1.77

^1^ Polymerization was initiated with AIBN (1 mol %) at 60 °C for 16 h in bulk or solvent (3.0 mL); ^2^ Determined by ^1^H NMR (400 MHz, CDCl_3_, 25 °C); ^3^ Determined by SEC (PS standards, THF, 40 °C); ^4^ Initiated by 2,2′-Azobis(4-methoxy-2,4-dimethylvaleronitrile).

**Table 2:** Radical (co)polymerizations of DMαHAA with DEAA and the cloud points of the resulting polymer.

Run ^1^	DMαHAA (mmol)	DEAA (mmol)	Yield (%)	Composition ^2^		*M*_n_^3^	*M*_w_/*M*_n_^3^	*T*_c_ (°C)
				DMαHAA	DEAA			
3	-	3.12	78	0	100	25,000	1.71	32
10	1.57	6.40	99	6	94	23,000	1.89	34
5	1.65	1.53	57	20	80	1400	2.90	44
11	3.12	1.60	55	40	60	4800	1.77	46
12	6.43	1.63	57	65	35	1700	2.63	64

^1^ Polymerization was initiated with AIBN (1 mol %) at 60 °C in 1,4-dioxane (3.0 mL) for 16 h; ^2^ Determined by ^1^H NMR (400 MHz, CDCl_3_, 25 °C); ^3^ Determined by SEC (PS standards, THF, 40 °C).

## Supplementary Materials

The following are available online at www.mdpi.com/2073-4360/8/10/374/s1, Video S1: Thermo-response of poly(DMαHAA-*co*-DEAA), obtained in Run 5, in water (1 wt %).
